# Sex, Race, and Ethnicity Differences Among Residents With Exceptionally High Graduate Medical Education Ratings

**DOI:** 10.1001/jamanetworkopen.2026.4017

**Published:** 2026-03-30

**Authors:** Jung G. Kim, Karen E. Hauer, Christy K. Boscardin, Jasmine I-Shin Su, Eric S. Holmboe, Lyuba Konopasek, Isabel L. Chen, Cristina M. Gonzalez, Gbenga G. Ogedegbe, Jesse Burk-Rafel, Mytien Nguyen, John S. Andrews, David D. Henderson, Judee Richardson, William McDade, Dowin Boatright

**Affiliations:** 1 Ronald O. Perelman Department of Emergency Medicine, New York University Grossman School of Medicine, New York; 2 Department of Medicine, University of California San Francisco, San Francisco; 3 Intealth, Philadelphia, Pennsylvania; 4 Department of Health Systems Science, Kaiser Permanente Bernard J. Tyson School of Medicine, Pasadena, California; 5 Department of Population Health, New York University Grossman School of Medicine, New York; 6 Department of Medicine, New York University Grossman School of Medicine, New York; 7 American Medical Association, Chicago, Illinois; 8 Department of Family Medicine, University of Connecticut School of Medicine, Farmington; 9 Rush University, Department of Anesthesiology, Chicago, Illinois

## Abstract

**Importance:**

Limited research exists on sex, racial, and ethnic disparities in required graduate medical education (GME) resident competency ratings across specialties during sensitive periods when career decision-making occurs. Rating disparities using an antideficit-based approach measured by exceptionally high ratings are underexplored in GME.

**Objective:**

To assess the association of exceptionally high ratings in the Accreditation Council for Graduate Medical Education (ACGME) Milestones during time-sensitive training periods across specialties with differences among residents’ characteristics, including sex, race, and ethnicity.

**Design, Setting, and Participants:**

This cross-sectional analysis was conducted between March 15 and December 31, 2025, using 2018 to 2021 Association of American Medical Colleges and ACGME data. Postgraduate year (PGY) 2 residents training at US ACGME-accredited emergency medicine, family medicine, internal medicine, obstetrics and gynecology, pediatrics, and surgery residency programs between 2018 and 2021 who self-reported sex, race, or ethnicity were studied.

**Exposure:**

Required Milestones ratings at the end of PGY-2 training associated with resident sex and race or ethnicity (underrepresented in medicine [URiM] and Asian), while controlling for preresidency Step 2 Clinical Knowledge examination scores.

**Main Outcomes and Measures:**

Proportion and adjusted odds ratios (AORs) for exceptionally high resident-level ratings (80th percentile level) across competencies in interpersonal and communication skills, medical knowledge, patient care, practice-based learning and improvement, professionalism, and systems-based practice.

**Results:**

Among 19 492 PGY-2 residents across 1754 programs, 10 384 (53.3%) were female, 28 (0.14%) American Indian or Alaskan Native, 4327 (22.2%) Asian, 1106 (5.7%) Black, 1008 (5.2%) Hispanic or Latinx, 3 (0.02%) Native Hawaiian or Pacific Islander, 12 269 (62.9%) White, 751 (3.9%) reporting 2 or more races, and 3423 (17.6%) classified as URiM. Exceptional rating differences were identified by sex, race, and ethnicity. Across all specialties, female residents had greater odds for 80th percentile ratings (AOR, 1.12; 95% CI, 1.05-1.21; *P* < .001); whereas when compared with White residents, URiM residents (AOR, 0.68; 95% CI, 0.62-0.76; *P* < .001) and Asian residents (AOR, 0.67; 95% CI, 0.60-0.74; *P* < .001) were less likely to have 80th percentile ratings than White residents. Within specialties, URiM residents in emergency medicine, family medicine, internal medicine, obstetrics and gynecology, and surgery were less likely to have 80th percentile ratings, whereas Asian residents in family medicine, internal medicine, pediatrics, and surgery were also less likely than White residents.

**Conclusion and Relevance:**

In this cross-sectional national study of residents, exceptionally higher ratings were associated with differing resident characteristics during crucial career planning phases. These results suggest the need for more studies to explore factors of resident success during GME training.

## Key Points

**Question:**

Are exceptionally high ratings on the Accreditation Council for Graduate Medical Education Milestones during time-sensitive periods when career decision-making occurs associated with residents’ characteristic differences in sex, race, and ethnicity across medical specialties?

**Findings:**

This cross-sectional study of 19 492 second-year residents across 1754 programs found that female residents were more likely to score in the 80th percentile or greater than male residents, while Asian residents and residents from groups underrepresented in medicine were less like than White residents to score that highly. Differences were also found among graduate medical education (GME) medical specialties.

**Meaning:**

These results suggest that differences in characteristics of residents who receive exceptionally high GME ratings during time-sensitive training periods may affect their career path decision-making and future opportunities.

## Teaser

This cross-sectional study of second-year residents examines the association between exceptionally high performance ratings on the Accreditation Council of GME Milestones and differences among resident sex, race, and ethnicity.

## Introduction

Increasing sex, racial, and ethnic representation within graduate medical education (GME) has long been argued to better address patient-specific needs, reflect communities’ concordance, and enrich professional training environments.^1,2,3,4^ However, differences in community-based representation across GME persist due to historical imbalances among sex, racially, and ethnically underrepresented in medicine (URiM) physicians nationally and by specialty.^5,6,7,8,9,10,11^ Studies that examine the persistent underrepresentation among physicians are nascent, yet disparities in their assessments during GME may contribute to this underrepresentation.^10,12,13^

Prior resident assessment bias studies have found that individual traits, including sex, race, and ethnicity, may overshadow assessment for actual clinical competency.^13,14,15^ These studies benchmarked rating disparities from averaged resident levels, which focus predictors on risk, struggle, and attrition. In contrast, studying exceptional levels of performance using an antideficit achievement framework is less common in GME.^16,17,18^ Use of an asset-based approach from prior studies^16,62^ in science, technology, engineering, and mathematics education may unveil markers of GME success among those historically marginalized to better understand factors that promote resilience and professional growth and whether these attributes translate to improved sex, racial, and ethnic representation in academic health settings.

Assessing resident performance is a key factor that informs resident promotion and guides career choices and decisions.^19,20^ However, early evidence found that URiM residents rated lower than their peers in early years of GME training and that these rating disparities emerge in the second postgraduate year (PGY-2).^13,15^ Studying PGY-2 residents for exceptionally high ratings is also important when distinctions are considered, including chief resident selection for some specialties, and for post-GME opportunities in academic health settings that offer career advancement and reputational prestige.^21,22,23,24,25,26,27,28,29^ Additionally, although resident performance assessments have been studied within individual specialties, they offer limited insight into GME as a whole and the systemic factors underlying sex, racial, and ethnic underrepresentation across academic health settings.^14,15,19^

To better understand the implications of GME rating differences during time-sensitive training periods and across multiple specialties, we investigated (1) the differences in exceptionally high performance ratings associated with resident characteristics and (2) whether these ratings are similar or different across specialties with higher or lower sex, racial, and ethnic resident representation. Using an antideficit achievement framework, we explored the association between exceptionally high performance ratings in the Accreditation Council of Graduate Medical Education (ACGME) Milestone ratings for all PGY-2 residents across 6 medical specialties in US-accredited ACGME training programs and differences among residents’ individual characteristics, including sex, race, and ethnicity.

## Methods

This study met exempt criteria from the New York University institutional review board as it did not involve human research participants and no informed consent was required. The study followed the Strengthening the Reporting of Observational Studies in Epidemiology (STROBE) statement.^30^

### Study Design and Data Sources

This cross-sectional study examined resident-level data from the Association of American Medical Colleges (AAMC) and ACGME for PGY-2 residents training in US ACGME-accredited residency training programs. Residency programs training residents in the specialties of emergency medicine, family medicine, internal medicine, obstetrics and gynecology, pediatrics, and surgery between July 1, 2018, and June 30, 2021, were examined because they represented the GME training programs with the highest number of programs; had known representation differences by sex, race, and ethnicity; and had national-level data available.^31,32^ PGY-2 residents were purposefully sampled based on selecting a harmonized period for varying lengths of GME training and prior research detecting early rating differences in GME during this period, including training programs’ familiarity with a resident’s performance after a full year of GME training and during a time-sensitive training period when residents in most GME specialties begin to prepare for post-GME career planning.^26,27,29,33^

Resident characteristics, including self-reported sex (female and male) and URiM status, were examined to explore systematic differences in ratings. URiM was categorized using the AAMC definition that includes any resident who self-identified as being historically racially and ethnically underrepresented for one or more of the following groups: American Indian or Alaska Native; Black or African American; Hispanic, Latino, or of Spanish origin; or Native Hawaiian or Other Pacific Islander.^34^ To adjust for potential baseline differences in resident medical knowledge before entering residency, US Medical Licensing Examination Step 2 Clinical Knowledge (CK) was included as a covariate in our analytic models. Due to the relatively low proportion of specific race and ethnic groups within URiM representation, we could not perform adjusted regression models using disaggregated racial and ethnic groups for American Indian and Alaskan Native, Hispanic and Latinx, and Native Hawaiian and Pacific Islander residents. Our analysis also excluded residents born outside the US because non-US citizens are not classified by specific race and ethnicity in the available dataset and represent more so their characteristics of training location origin vs their actual race and ethnicity.

To identify exceptionally high performance, we used the positive deviance approach to operationalize the antideficit achievement conceptual framework. This method examines widely accepted, empirical measures on established criteria for recognizing promising individuals compared with their peers and measured by high percentile levels (80th percentile or greater).^16,35,36,37^ We quantified positive deviance through percentiles to determine a ranked order derived from the ACGME Milestone ratings. The ACGME requires all US-accredited GME training programs to evaluate residents on their core physician competencies in interpersonal and communication skills, medical knowledge, patient care, practice-based learning and improvement, professionalism, and systems-based practice to qualify them for unsupervised practice, as reported by the ACGME Milestones system.^38,39,40^ Positive deviance analysis was performed to identify high-performing residents rated by their ACGME Milestones, using total scores (scale of 0-9, with 0 indicating poor performance across competencies and 9 indicating high performance across competencies) received at the end of the PGY-2 training year.^15,19^

### Statistical Analysis

To determine exceptionally high performance ratings for residents while standardizing Milestone ratings across multiple specialties, we clustered all resident-level ratings by each GME training program, log-transformed to determine a ranked normal distribution for each training program, and then coded for high performance (yes or no) if the resident’s Milestone score was at the 80th percentile or higher compared with their training program peers. Our working definition of high performance specified percentile cutoffs at the 80th percentile to reflect high performance at the top quintile in a program and examine a resident’s potential to perform above their peers. We also conducted sensitivity analyses at the 90th percentile to model very high performance and used an additional percentile cutoff based on past literature in positive deviance.^38^ To contextualize the Milestones distributions for each specialty, the mean (SD) percentiles for each specialty were as follows: emergency medicine, 61.4 (18.5); family medicine, 63.1 (18.8); internal medicine, 61.8 (19.5); obstetrics and gynecology, 56.8 (21.3); pediatrics, 62.8 (18.9); and surgery, 58.9 (21.1).

We examined the frequency, central tendency, and variation of Milestones’ positive deviance and descriptively analyzed their associations with resident sex, race or ethnicity status, and Step 2 CK scores that were aggregated across all GME specialties sampled then by each GME specialty independently. To determine whether sex and aggregated race or ethnicity groups (URiM and Asian) were associated with positive deviance in PGY-2 Milestone ratings, we performed multivariable logistic regression that calculated adjusted odds ratios (AORs) with 95% CIs at the 80th percentile ratings for all specialties aggregated and then for each specialty independently. Sex (male as the reference group) and Asian and URiM groups (White as the reference group) were our main variables, with Step 2 CK scores as covariates to account for baseline medical knowledge. We then repeated multivariable logistic regression models at the 90th percentile ratings for our sensitivity analyses.

Stata, version 19.0 (StataCorp) and R Studio, version 2025.09.0 + 387 (R Foundation for Statistical Computing) were used to conduct analyses between March 15 and December 31, 2025. A 2-sided *P* < .05 was considered statistically significant.

## Results

Among 19 492 PGY-2 residents training in 1754 US ACGME-accredited emergency medicine, family medicine, internal medicine, obstetrics and gynecology, pediatrics, and surgery programs, 10 384 (53.3%) were female, 9108 male (46.7%), 28 (0.14%) American Indian or Alaskan Native, 4327 (22.2%) Asian, 1106 (5.7%) Black, 1008 (5.2%) Hispanic or Latinx, 3 (0.02%) Native Hawaiian or Pacific Islander, 12 269 (62.9%) White, 751 (3.9%) reporting 2 or more races, and 3423 (17.6%) URiM. The mean (SD) Step 2 CK score was 243.5 (15.0). Statistically significant differences in exceptionally high ratings were not found when examining all specialties (Table).

**Table.** Milestone Ratings at the 80th Percentile Levels by Specialty, Sex, Race, and USMLE Step 2 CK Scores

Variable	80th Percentile, No. (%)^a^		Total (N = 19 492)	*P* value
	Yes (n=3799)	No (n=15 693		
Specialty				
Emergency medicine	589 (15.5)	2469 (15.7)	3058 (15.7)	.07
Family medicine	563 (14.8)	2290 (14.6)	2853 (14.6)	
Internal medicine	1256 (33.1)	5329 (34.0)	6585 (33.8)	
Obstetrics and gynecology	386 (10.2)	1545 (9.9)	1931 (9.9)	
Pediatrics	672 (17.7)	2513 (16.0)	3185 (16.3)	
Surgery	333 (8.8)	1547 (9.9)	1880 (9.6)	
Sex				
Male	1696 (44.4)	7412 (47.2)	9108 (46.7)	.004
Female	2103 (55.4)	8281 (52.8)	10 384 (53.3)	
Race				
American Indian or Alaskan Native	4 (0.1)	24 (0.2)	28 (0.1)	<.001
Asian	660 (17.4)	3667 (23.4)	4327 (22.2)	
Black	113 (3.0)	993 (6.3)	1106 (5.7)	
Hispanic or Latinx	173 (4.6)	835 (5.3)	1008 (5.2)	
Native Hawaiian or Pacific Islander	0	3 (0.02)	3 (0.02)	
White	2707 (71.3)	9562 (60.9)	12 269 (62.9)	
≥2 Races	142 (3.7)	609 (3.9)	751 (3.9)	
Underrepresented in medicine^b^	514 (13.5)	2909 (18.5)	3423 (17.6)	<.001
USMLE Step 2 CK score, mean (SD)	245.9 (14.7)	242.9 (14.9)	243.5 (15.0)	<.001

Abbreviation: USMLE Step 2 CK, US Medical Licensing Examination Step 2 Clinical Knowledge.

^a^ Unless otherwise indicated.

^b^ Underrepresented in medicine is a separate categorical variable constructed using racial and ethnic data. It includes American Indian or Alaska Native, Black, Hispanic or Latinx, Native Hawaiian or Pacific Islander, and multiracial individuals.

### Differences by Sex

By sex, more females were rated with positive deviance than males, with higher proportions for females at the 80th percentile (2103 females [55.4%] vs 1696 males [44.4%], *P* = .004). Multivariable logistic regression models examining sex and all specialties together while adjusting for race, ethnicity, and Step 2 CK scores found that females had higher odds than males for ratings at the 80th percentile (AOR, 1.12; 95% CI, 1.05-1.21; *P* < .001). When each specialty was examined independently, female residents in internal medicine programs had 20% higher odds for rating at the 80th percentile (AOR, 1.20; 95% CI, 1.07-1.38; *P* = .002) (Figure).

**Figure.**
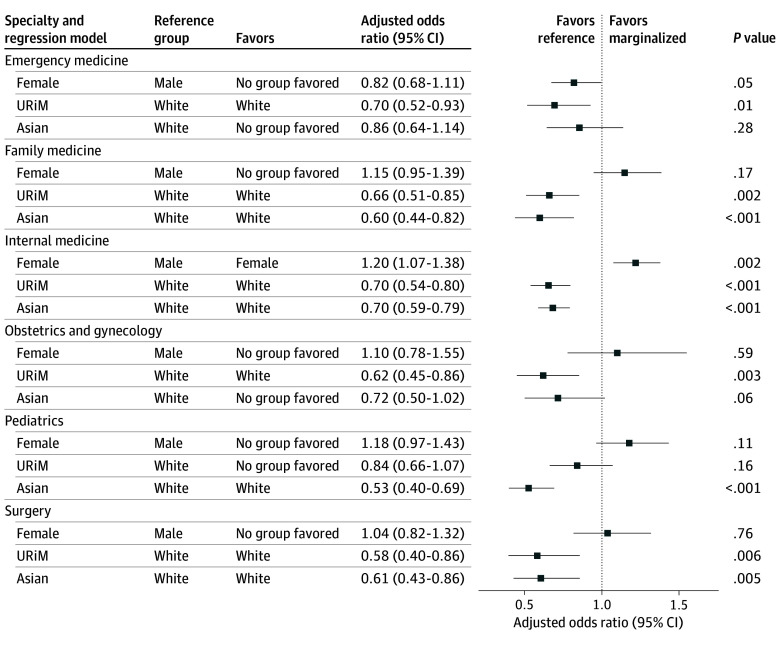
Forest Plot of the Adjusted Odds Ratios for Exceptionally High Performance Ratings During Graduate Medical Education by Sex, Racial, and Ethnic Groups Across Multiple Specialties URiM indicates underrepresented in medicine and includes American Indian or Alaska Native, Black, Hispanic or Latinx, Native Hawaiian or Pacific Islander, and multiracial individuals.

### Differences by Race and Ethnicity

We also found differences when comparing positive deviant rating level (80th percentile) across all specialties by disaggregated race and ethnic groups: American Indian and Alaskan Native (4 [0.1%]), Asian (660 [17.4%]), Black (113 [3.0%]), Hispanic and Latinx (173 [4.6%]), Native Hawaiian and Pacific Islander (0), White (2707 [71.3%]), and residents reporting 2 or more races (142 [3.7%]). Residents who identified as URiM were less likely to have exceptionally higher ratings (514 [13.5%], *P* < .001) than those identifying as non-URiM.

In adjusted logistic regression models that aggregated all specialties and adjusted for sex and Step 2 CK scores, URiM (AOR, 0.68; 95% CI, 0.62-0.76; *P* < .001) and Asian (AOR, 0.67; 95% CI, 0.60-0.74; *P* < .001) residents compared with White residents had lower odds for 80th percentile ratings. For models examining each specialty independently (Figure), URiM compared with White residents had lower odds for exceptionally higher ratings at 80th percentile in emergency medicine (AOR, 0.70; 95% CI, 0.52-0.93; *P* = .01), family medicine (AOR, 0.66; 95% CI, 0.51-0.85; *P* = .002), internal medicine (AOR, 0.70; 95% CI, 0.54-0.80; *P* < .001), obstetrics and gynecology (AOR, 0.62; 95% CI, 0.45-0.86; *P* = .003), and surgery (AOR, 0.58; 95% CI, 0.40-0.86; *P* = .006). Asian residents compared with White residents had lower odds for exceptionally high ratings in family medicine (AOR, 0.60; 95% CI, 0.44-0.82; *P* < .001), internal medicine (AOR, 0.70; 95% CI, 0.59-0.79; *P* < .001), pediatrics (AOR, 0.53; 95% CI, 0.40-0.69; *P* < .001), and surgery (AOR, 0.61; 95% CI, 0.43-0.86; *P* = .005).

Sensitivity analyses estimating very high performance at the 90th percentile for all specialties found no difference in sex for ratings after adjusting for race, ethnicity, and Step 2 CK scores; URiM with lower odds than White residents after adjusting for sex and Step 2 (AOR, 0.74; 95% CI, 0.64-0.85; *P* < .001); and Asian with lower odds than White residents after adjusting for sex and Step 2 CK scores (AOR, 0.66; 95% CI, 0.50-0.76; *P* < .001). For models examining each specialty independently (eFigure in Supplement 1), URiM residents had lower odds for 90th percentile ratings than White residents in internal medicine programs (AOR, 0.60; 95% CI, 0.46-0.80; *P* < .001) and obstetrics and gynecology (AOR, 0.57; 95% CI, 0.35-0.93; *P* = .02). Asian residents compared with White residents had lower odds at the 90th percentile levels in family medicine (AOR, 0.64; 95% CI, 0.42-0.99; *P* = .046), internal medicine (AOR, 0.70; 95% CI, 0.56-0.84; *P* < .001), pediatrics (AOR, 0.59; 95% CI, 0.40-0.85; *P* = .005), and surgery (AOR, 0.42; 95% CI, 0.23-0.77; *P* = .01) (eFigure in Supplement 1).

## Discussion

In this cross-sectional study using an antideficit achievement framework across multiple GME specialties, we found that, after accounting for pre-GME medical knowledge (Step 2 CK scores), sex and race and ethnic groups were associated with differences in exceptionally high ratings during critical GME training periods. Within each specialty, greater differences were found for females training in internal medicine, who had notably higher odds for ratings at the 80th percentile compared with males. In contrast, URiM and Asian residents were less likely to receive exceptionally high ratings across all adjusted analytic models. This study is the first, to our knowledge, to highlight that sex, race, and ethnicity remain associated with differences in required training ratings when examining multiple GME specialties concurrently.

The differences in ratings by sex, with more females rated exceptionally high, contrast with prior Milestone research that found lower ratings based on the resident characteristic of sex.^41^ Other Milestone studies^13,15,41,42,43,44,45^ in emergency medicine, ophthalmology, and surgery reported that females had lower than expected training score levels or lower mean Milestone ratings at different periods in their training, whereas another study^41^ in internal medicine alone found little difference. Our findings with sex-based differences within Milestones ratings offer new insight into exceptionally high GME performance. Despite being rated at the 80th percentile and represented in our sample overall, further study is needed to better understand why female physicians with demonstrated exceptional GME performance remain underrepresented in positions of leadership in academic health settings and among peer-reviewed journal editors.^46,47,48^

This multispecialty study also extends the findings of prior national GME Milestone studies.^13,15,41,42,43,44,45^ Prior studies^13,14,15^ in emergency and internal medicine have reported that URiM residents were rated lower in program-level mean core competency ratings than non-URiM residents, even at GME graduation. Our study, by examining exceptionally high ratings using an antideficit framework, highlights the potential to study successful URiM residents from historically marginalized backgrounds and whose success has been understudied. Furthermore, unlike prior studies^13,15,41,42,43,44,45^ that focused on independent medical specialties, this study identifies systematic rating differences when sampling most of the major GME medical specialties and when accounting for standardized medical knowledge scores from residents’ medical school performance before beginning GME. This finding calls for additional studies to better understand our findings on URiM and Asian residents with reported lower odds for exceptionally higher ratings across multiple specialties, despite controlling for baseline medical knowledge (Step 2 CK). Our study’s indication of possible biases also underscores the need to examine other factors within each training program’s GME assessment system that could promote fair, objective, and asset-based assessments for residents to learn and advance in GME, while also recognizing potential leaders. This finding calls for further examination to ensure a training program’s assessment system provides equitable opportunities and unbiased measures, which are iteratively calibrated using continuous quality improvement efforts to promote rating fairness and to use these systems for opportunities to identify future academic leaders.^49^

Our results also raise important questions regarding the implications of Asian residents receiving less exceptionally high ratings in GME. Despite being fairly represented among medical schools and in GME, other reports on Asian physicians indicate that they represent less than 21% of academic health faculty positions (even less in academic health leadership positions at 8.3%) and historically have had no representation as medical school deans.^48,50^ Our findings may provide additional context with other reports and suggest that the absence of exceptionally high ratings among Asian residents dissuades their pursuit of leadership positions in academic health settings. For URiM residents, current pathway efforts aim to increase their representation in medical schools and subsequent increase in representation within GME, academic health faculty placements, and academic health leadership positions. Our study’s findings on URiM and Asian residents raise the possibility that the lower odds of these individuals being rated exceptionally high may hinder these existing efforts.

Importantly, this study’s antideficit achievement conceptual framework and positive deviance analyses identify exceptionally high performance among historically marginalized groups, with possible inferences to their potential as leaders. Findings could help increase community-concordant representation among academic leadership positions. Our results also inform future studies that could explore nuanced factors of success for the female, URiM, and Asian residents identified as highly rated in our study. These studies include analyses on the intersectionality of sex with race and ethnicity, which studies with larger samples could explore. Understanding how these residents leveraged success to navigate GME training and how those attributes could catalyze the pursuit of leadership opportunities is critical in light of persistent underrepresentation in academic leadership among female, URiM, and Asian physicians.^51,52^ Identifying and sustaining these efforts are essential to advance the field of assessment disparities research that increases community-concordant representation in academic health settings and leadership roles.^51,52,53,54,55,56^

### Limitations

This study has several limitations. Sampling multiple specialties, which in themselves have differing characteristics (eg, internal medicine and its subspecialties), may mask intraspecialty factors and their approaches to clinical competence performance ratings, despite our use of a harmonized period with standardized Milestone ratings. Other factors include some specialties that are considered more competitive based on available positions and applicant volumes or specialties that have more dominant resident characteristics, which could perpetuate biases in evaluation ratings scored by their training program’s faculty. Relatedly, the presence of non-US international medical graduates, who account for one-quarter of GME residents and often fill empty GME slots after the GME match, should be explored. Future studies could examine the origin of training location and recategorize these individuals differently from their race and ethnicity characteristics.^57,58^ Furthermore, examining individual identities by aggregated race and ethnic groups fails to consider characteristics that are historically underrepresented (eg, rural backgrounds and first-generation physician).

We also could not directly account for the GME program–based rating factors that determine Milestones scores within training programs and that may be subjected to specific rater harshness characteristics due to the lack of available data. This issue calls for further research to examine GME program–level factors and more clarity regarding committee composition and training program criteria standards used by clinical competency committees in determining required Milestones ratings, including medical knowledge.^59,60^ Incorporating additional psychometric reliability methods that detect rating biases could lead to better understanding of the potential impact of Milestones scores received during sensitive training times in GME and their subsequent association with career opportunities and attainment of leadership positions.^61^

## Conclusions

This cross-sectional study found that female residents were more likely than male residents to achieve exceptionally high ratings across expected physician core competencies during time-sensitive GME training periods, while Asian residents and those from URIM groups were less likely than White residents to achieve exceptionally high scores, with differences also found among GME medical specialties. Our findings suggest the need for further research to test interventions that explore factors contributing to resident success and to explore the temporal associations between competency-based ratings received in GME and postresidency career placements.

## Notes

Supplement 1. **eFigure.** Forest Plot Showing Odds Ratio of Exceptionally High Resident-Level Ratings at the 90th Percentile Level

Supplement 2.
Data Sharing Statement
