# Supplementary material for: Sex, Race, and Ethnicity Differences Among Residents With Exceptionally High Graduate Medical Education Ratings
Source: JAMA Netw Open. 2026 Mar 30;9(3):e264017. doi: 10.1001/jamanetworkopen.2026.4017 (PMC13036576; doi:10.1001/jamanetworkopen.2026.4017)
Supplement: Supplement 1. — eFigure. Forest Plot Showing Odds Ratio of Exceptionally High Resident-Level Ratings at the 90th Percentile Level [file jamanetwopen-e264017-s001.pdf]

Supplemental Online Content

Kim JG, Hauer KE, Boscardin CK, et al. Demographic differences among residents with exceptionally high graduate medical education ratings. *JAMA Netw Open*. 2026;9(3):e264017. doi:10.1001/jamanetworkopen.2026.4017

eFigure. Forest Plot Showing Odds Ratio of Exceptionally High Resident-Level Ratings at the 90th Percentile Level

This supplemental material has been provided by the authors to give readers additional information about their work.

© 2026 Kim JG et al. *JAMA Network Open.*

eFigure 1. Forest Plot Showing Odds Ratio of Exceptionally High Resident-Level Ratings at the 90th Percentile Level

Specialty	Regression model	Reference group	Favors X/Y	Odds Ratio (95% CI)	p-value
Emergency Medicine	Female	Male	No group favored	0.94 (0.73–1.21)	0.65
	URiM	White	No group favored	0.70 (0.47–1.03)	0.066
	Asian	White	No group favored	0.69 (0.46–1.03)	0.068
Family Medicine	Female	Male	No group favored	1.13 (0.87–1.46)	0.37
	URiM	White	No group favored	0.85 (0.60–1.19)	0.33
	Asian	White	White	0.64 (0.42–0.99)	0.046
Internal Medicine	Female	Male	No group favored	1.01 (0.94–1.31)	0.2
	URiM	White	White	0.60 (0.46–0.80)	<.001
	Asian	White	White	0.70 (0.56–0.84)	<.001
Obstetrics & Gynecology	Female	Male	No group favored	1.02 (0.63–1.66)	0.92
	URiM	White	White	0.57 (0.35–0.93)	0.023
	Asian	White	No group favored	0.75 (0.45–1.24)	0.26
Pediatrics	Female	Male	No group favored	1.16 (0.89–1.52)	0.26
	URiM	White	No group favored	0.98 (0.71–1.33)	0.88
	Asian	White	White	0.59 (0.40–0.85)	0.005
Surgery	Female	Male	No group favored	1.09 (0.77–1.55)	0.63
	URiM	White	No group favored	0.83 (0.49–1.40)	0.48
	Asian	White	White	0.42 (0.23–0.77)	0.01

URiM: Underrepresented

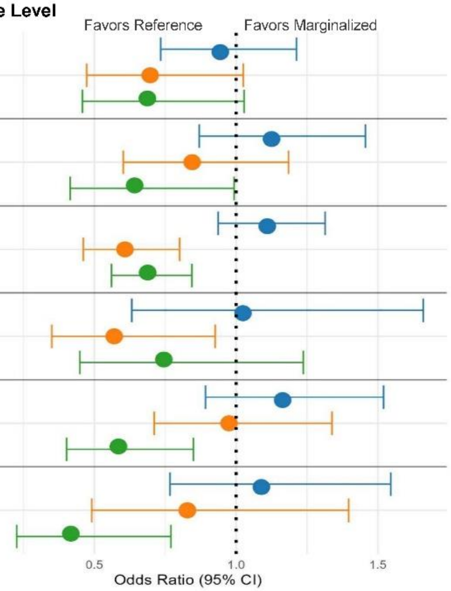

© 2026 Kim JG et al. *JAMA Network Open.*
