# Supplementary material for: Sex, Race, and Ethnicity Differences Among Residents With Exceptionally High Graduate Medical Education Ratings
Source: JAMA Netw Open. 2026 Mar 30;9(3):e264017. doi: 10.1001/jamanetworkopen.2026.4017 (PMC13036576; doi:10.1001/jamanetworkopen.2026.4017)
Supplement: Supplement 2. — Data Sharing Statement [file jamanetwopen-e264017-s002.pdf]

Data Sharing Statement

Kim. Sex, Race, and Ethnicity Differences Among Residents With Exceptionally High Graduate Medical Education Ratings. *JAMA Netw Open*. Published March 30, 2026.
doi:10.1001/jamanetworkopen.2026.4017

Data
Data available: No

Additional Information
Explanation for why data not available: DUA prevents sharing of data
